# Comparison of Masticatory Efficiency and Patient Satisfaction of Injection-Molded Conventional Complete Dentures and Biofunctional Prosthetic System Dentures: A Parallel Randomized Clinical Trial

**DOI:** 10.7759/cureus.42564

**Published:** 2023-07-27

**Authors:** Shankar Piramanayagam Vivek, Sivanesan Karthikeyan Jagdish, Krishnan Murugesan, John Peter, Chithambaradhas Sivakala Arunkumar, Balasubramaniam Muthukumar

**Affiliations:** 1 Department of Prosthodontics and Implantology, SRM Dental College and Hospital, Chennai, IND; 2 Department of Prosthodontics and Implantology, Chettinad Dental College and Research Institute, Kelambakkam, IND; 3 Department of Prosthodontics and Implantology, Meenakshi Ammal Dental College, Chennai, IND

**Keywords:** randomized trial, patient satisfaction, masticatory efficiency, denture adjustments, complete denture, biofunctional prosthetic system

## Abstract

Introduction

Complete loss of natural teeth severely compromises the masticatory efficiency of geriatric patients and directly affects their general health. Biofunctional prosthetic system (BPS) has been developed as an alternate method of denture fabrication to match the higher expectations of patients. Studies comparing BPS dentures with injection-molded conventional complete dentures (IM-CCD) are lacking. Our study aimed to compare the masticatory efficiency and patient satisfaction of BPS dentures with IM-CCD.

Methods

This trial was designed as a randomized, prospective, single-center, double-blinded study with two parallel arms (BPS group and IM-CCD group) having an equal allocation (1:1). Completely edentulous patients aged 45-80 who visited our outpatient department between January and August 2018 were screened and enrolled according to our inclusion and exclusion criteria. Random sequence generation was done using an online randomization program. Allocation concealment was done using sequentially labeled opaque envelopes. The participants and the outcome assessors were blinded. A single operator performed all the clinical procedures in both groups under the guidance of the instructor. The age and gender of the patients were recorded for demographic data at baseline. Primary outcomes were assessed six weeks after denture insertion. The number of denture adjustments required during the first six weeks after denture delivery was recorded as the secondary outcome. Masticatory efficiency was evaluated by the volumetric single-sieve method. An abbreviated version of the Oral Health Impact Profile questionnaire for edentulous patients (OHIP-EDENT) was used for patient satisfaction scores. The total OHIP-EDENT score was calculated by adding all the responses to the 19 questions (ranging from 0 to 38). Individual domain scores were calculated by adding the response to all the questions in that domain. All the data obtained were tested for normality using the Kolmogorov-Smirnov test. Data were analyzed using either unpaired Student’s T-test or Mann-Whitney U test for normal and non-normal data, respectively. The gender characteristics of the sample were compared using Chi-Square test.

Results

Two patients in the IM-CCD group and one patient in the BPS group were lost to follow-up. Hence only the secondary outcome data were analyzed for these patients. Primary outcome data of patients who were lost to follow-up were excluded from the analysis. No significant differences (p>0.05) were found between the two groups for age and gender characteristics of the samples. Masticatory efficiency for both peanut and carrot was found to be significantly higher (p<0.05) in the BPS group than in the IM-CCD group. No significant differences (p>0.05) in the mean scores were observed between the two groups for total OHIP-EDENT scores or the individual domain scores. No significant differences (p>0.05) were observed between the groups for the number of denture adjustments done.

Conclusion

Within the limitations of the trial, it can be concluded that the BPS dentures significantly improved the masticatory efficiency for both hard (carrots) and soft (peanuts) foods compared to the IM-CCD. However, there was no difference between the masticatory-related complaints domain scores between the two dentures. No significant differences were found between BPS dentures and IM-CCD with respect to overall patient satisfaction scores or post-insertion denture adjustments.

## Introduction

Complete loss of natural teeth severely compromises the masticatory efficiency of geriatric patients and directly affects their general health [1]. Complete dentures significantly improve the masticatory muscle activity, mandibular movements, and masticatory efficiency of edentulous patients [2]. Studies comparing masticatory efficiency and patient satisfaction in complete dentures with different occlusal schemes [3-5], posterior tooth forms [6], and impression techniques [7] have yielded conflicting results. In the consensus statement on complete denture occlusion, Goldstein et al. [8] concluded that chewing ability and patient satisfaction were not influenced by the posterior tooth forms or occlusal scheme. Jayaraman et al. [9] found no association between various complete denture impression techniques and patient satisfaction. Soboleva and Rogovska [10] reported that overall patient satisfaction and comfort with complete dentures were found to be associated with the stability of the mandibular dentures rather than other factors.

Biofunctional prosthetic system (BPS) has been developed as an alternate method of denture fabrication to match the higher expectations of patients [11]. The BPS protocol uses a modified impression technique using the “Accudent Impression System” (AccuDent XD Impression System, Ivoclar Vivadent, Liechtenstein), “Gnathometer M” which is a type of intra-oral tracing device, and injection molding technique for denture processing [11]. Few studies have shown that centric records obtained using intra-oral tracers were more accurate compared to other methods [12,13]. Dentures processed using the injection molding technique had better adaptation and lesser processing errors [14,15]. A long-term study by Xhajanka et al. [16] reported better denture stability and lesser decubitus with BPS dentures compared to conventional complete dentures (CCD). Madana Gopal et al. [17] found that BPS dentures could reduce the stresses on the oral tissues compared to CCD by inducing changes in salivary amylase and cortisol levels. Matsuda et al. [18] reported no differences in patient satisfaction and denture adjustments between BPS and CCD. However, Khazi et al. [19] stated that a large number of clinical trials are still required to establish the superiority of BPS over conventional dentures.

Studies comparing BPS dentures with injection-molded conventional complete dentures (IM-CCD) are lacking. Our study aimed to compare the masticatory efficiency and patient satisfaction of BPS dentures with IM-CCD. Primary outcome measures were masticatory efficiency and patient satisfaction. The secondary outcome measure was the number of post-insertion denture adjustments. The null hypothesis stated that there would be no difference in masticatory efficiency, patient satisfaction, and post-insertion denture adjustments between BPS dentures and IM-CCD.

## Materials and methods

This trial was designed as a randomized, prospective, single-center, double-blinded study with two parallel arms (BPS group and IM-CCD group) having an equal allocation (1:1). The trial protocol and consent forms were approved by the institutional ethics committee (IRB approval No: SRMDC/IRB/2017/MDS/No.207). The trial was registered in the Clinical Trials Registry of India (CTRI No: CTRI/2020/01/023007). No deviations were made from the registered protocol. The clinical trial was conducted following the Declaration of Helsinki and Consolidated Standards of Reporting Trials (CONSORT) guidelines. Written consent was obtained from all the participants.

Sample size and study participants

The sample size was estimated using statistical software (G*Power, Version 3.1, Düsseldorf, Germany). The sample size of 34 (17 per group) was obtained using a presumed effect size of 0.5, alpha error of 5%, and 20% beta error. The final sample size (N) was increased to 40 (20 per group) to adjust for a 15% dropout [20]. Completely edentulous patients aged 45-80 who visited our outpatient department between January and August 2018 were screened and enrolled according to our inclusion and exclusion criteria (Table 1). Only ideal or minimally compromised patients with well-formed ridges and normal ridge relations were included in the trial.

**Table 1:** Inclusion and exclusion criteria TMJ: temporomandibular joint.

Criteria used for enrollment of patients	
Inclusion criteria	1. Patients between 45 and 80 years of age
	2. Healthy adult patients
	3. Patients who were self-motivated to wear dentures
	4. First-time denture wearers
	5. Well-formed residual alveolar ridges
Exclusion criteria	1. Patients with indifferent or hysterical attitudes
	2. Debilitating systemic diseases
	3. TMJ disorders
	4. Diseases of bones and joints
	5. Oral mucosal diseases
	6. Neurological/psychiatric/cognitive disorders
	7. Knife edge ridge/flabby ridges/tori or undercuts
	8. Smoking, pan chewing
	9. Parafunctional habits
	10. Xerostomia/hypersalivation
	11. Restricted tongue movement
	12. Palatal defects
	13. Hyperactive mentalis muscle
	14. Very high frenal attachments

Randomization, allocation concealment, and blinding

Random sequence generation was done by one of the investigators (B.M) using an online randomization program (https://www.randomizer.org/#randomize). Allocation of participants to either the BPS group (N=20) or the IM-CCD group (N=20) was done by another investigator (K.M) using the lot method. Allocation concealment was done using sequentially labeled opaque envelopes. Both these investigators (B.M and K.M) were not involved in the clinical procedures, outcome assessment, or data analysis. The type of denture to be fabricated for each patient was revealed to the operator (S.P.V) and the clinical instructor (S.K.J) only after allocation. The participants and the outcome assessors (C.S.A and J.P) were blinded for the type of denture delivered to the patient. Blinding was done by avoiding visual differences between the BPS and IM-CCD dentures (no logos were used on BPS dentures). Care was taken to make the wax-up, processing, and finishing to be identical between the two groups.

Fabrication of BPS dentures

**Figure 1:**
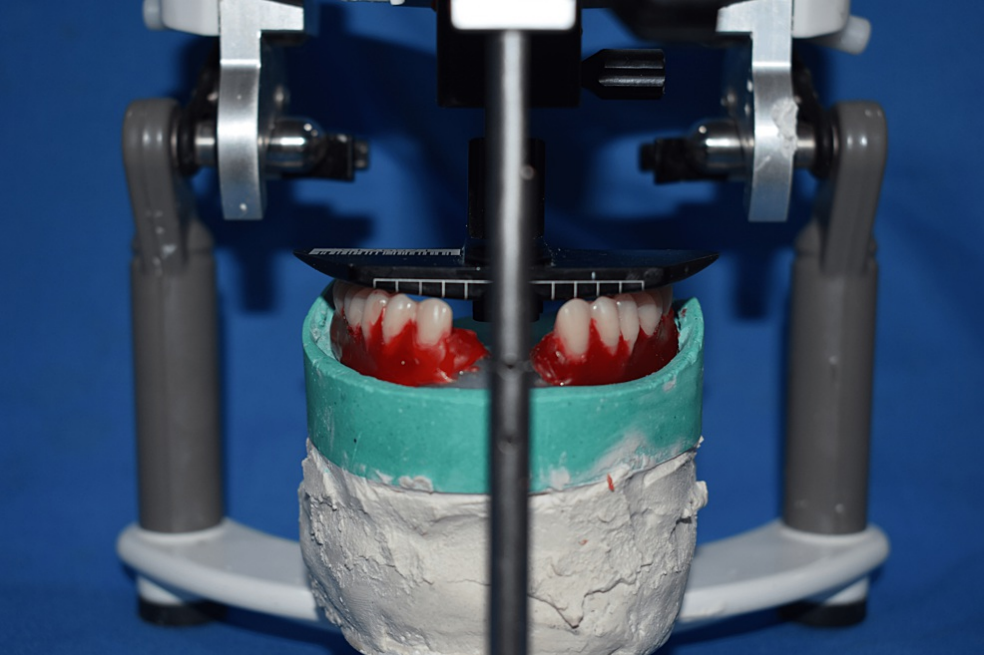
Template for the arrangement of teeth in BPS dentures BPS: biofunctional prosthetic system.

A single operator (S.P.V) performed all the clinical procedures in both groups under the guidance of the instructor (S.K.J). In the BPS group, maxillary and mandibular primary impressions were made using BPS impression trays (AccuDent Trays, Ivoclar Vivadent, Liechtenstein) using high and low viscosity alginate (AccuDent XD Impression System, Ivoclar Vivadent, Liechtenstein). Vertical dimensions at rest and occlusion were determined using the Niswonger method [21]. The vertical dimension at rest was determined by measuring the distance between the tip of the nose and the chin at the mandibular rest position. The vertical dimension of occlusion was calculated by reducing 3 mm of freeway space from the vertical dimension at rest. Tentative centric relation was recorded using the bite registration tray (Centric Tray, Ivoclar Vivadent, Liechtenstein) at the vertical dimension of occlusion using tray viscosity alginate (AccuDent XD Impression System, Ivoclar Vivadent, Liechtenstein). The patient was guided to the centric position using the bimanual manipulation technique [22]. Maxillary and mandibular casts were mounted on an articulator (Stratos 100, Ivoclar Vivadent, Liechtenstein) using the tentative centric record. Bite rims were fabricated using visible light cure tray material (Poly Tray, Delta Labs, India), and Gnathometer “M” was attached. Border molding was done using heavy body impression material (Virtual Heavy body, Ivoclar Vivadent, Liechtenstein), and closed mouth impressions were made using light body material (Virtual light body, Ivoclar Vivadent, Liechtenstein). Plastic plates on the Gnathometer “M” were removed to record the centric relation, and the tracing plates were attached. The centric record was then obtained by using hard setting addition silicone (Virtual, Ivoclar Vivadent, Liechtenstein), and casts were mounted on a semi-adjustable articulator (Stratos 100, Ivoclar Vivadent, Liechtenstein). The height of the maxillary and mandibular occlusal rims was determined by dividing the inter-vestibular distance into equal halves [11,16-18]. Teeth arrangement was done with the help of teeth setting template (Figure 1) using semi-anatomic teeth (Ivostar-Gnathostar, Ivoclar Vivadent, Liechtenstein) according to BPS principles [11,16-18]. Wax try-in was done in the third appointment. Processing was done by injection molding technique using the high-impact resin (SR Ivocap, Ivoclar Vivadent, Liechtenstein). BPS logos were not incorporated in dentures fabricated using the BPS protocol. Dentures were trimmed, polished, and delivered to the patient in the fourth appointment.

Fabrication of IM-CCD

For the IM-CCD group, primary impressions were made using irreversible hydrocolloid (Zelgan Plus, Dentsply India Pvt Ltd, India), and primary casts were obtained. Custom trays were fabricated using visible light cure tray material (Poly Tray, Delta Labs, India), and border molding was done using a low-fusing compound (DPI Pinnacle, India). Secondary impressions were made using zinc oxide eugenol impression paste (DPI Impression Paste, Dental Products of India, India). Wax bite rims were fabricated on light cure tray material (Poly Tray, Delta Labs, India). Maxillomandibular relations were established using the Niswonger method [21]. The vertical dimension at rest was determined by measuring the distance between the tip of the nose and the chin at the mandibular rest position. The vertical dimension of occlusion was calculated by reducing 3 mm of freeway space from the vertical dimension at rest. The occlusal rims were adjusted to the vertical dimension of occlusion, and the mandible was guided to the centric position using the bimanual manipulation technique [22]. Centric relation was recorded at the vertical dimension of occlusion using silicone bite registration paste (Virtual CAD Bite, Ivoclar Vivadent, Liechtenstein). An indirect facebow transfer was done using the springbow (Hanau Springbow, Whip Mix Corporation, USA). Casts were mounted on a semi-adjustable articulator (Hanau Wide-Vue, Whip Mix Corporation, USA) using the centric record. Teeth arrangement was done according to the conventional principles [17] using semi-anatomic teeth (Ivostar-Gnathostar, Ivoclar Vivadent, Liechtenstein). Try-in of the denture was done in the fourth appointment. Care was taken to make the wax-up identical to the BPS group to avoid any visual differences. Dentures were processed by injection molding technique using the high-impact resin (SR Ivocap, Ivoclar Vivadent, Liechtenstein). Dentures were trimmed, polished, and delivered to the patients in the fifth appointment.

Outcome assessment

**Figure 2:**
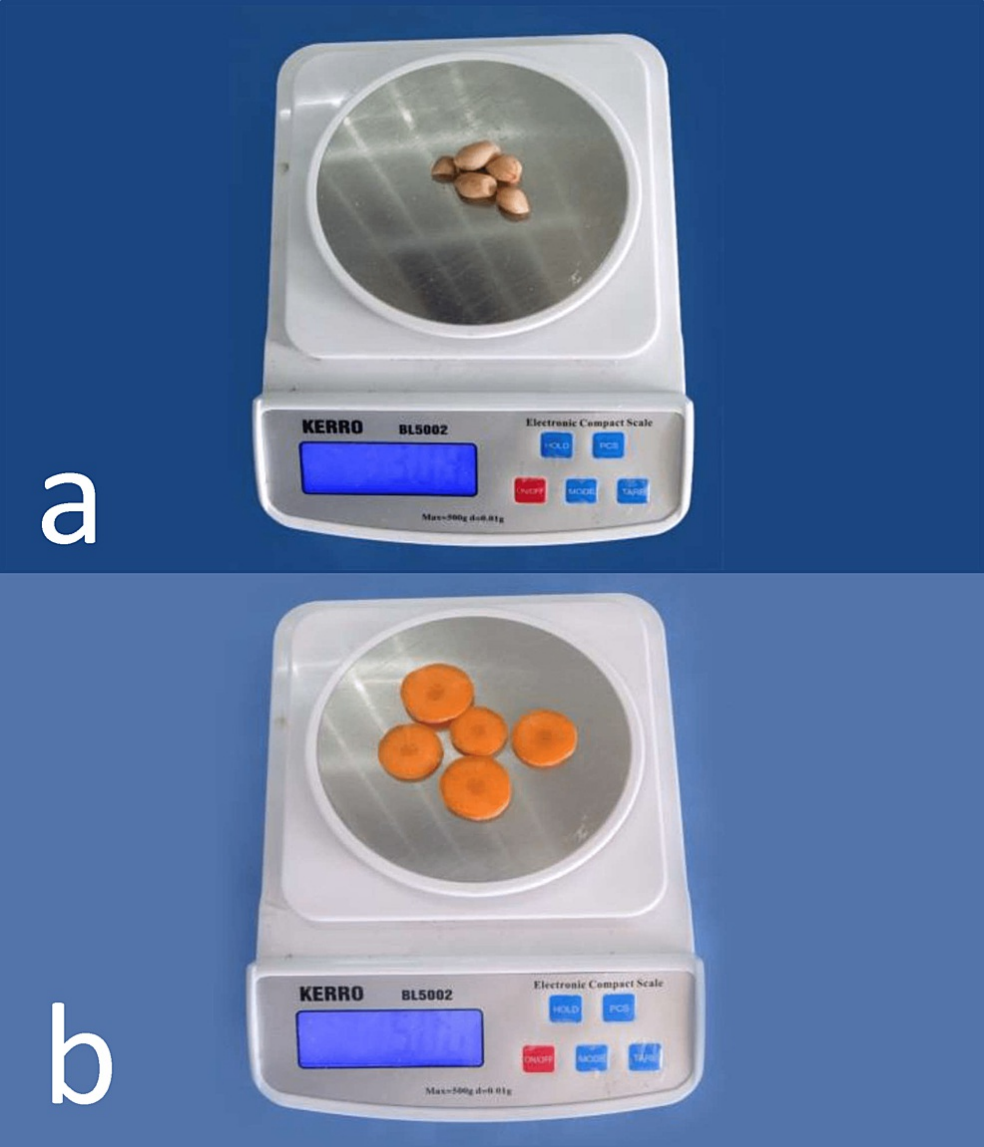
Weighing of test foods a) Boiled peanuts (soft food), and b) carrots cut into disks (hard food).

The age and gender of the patients were recorded for demographic data at baseline. Primary outcomes were assessed six weeks after denture insertion. The number of denture adjustments required during the first six weeks after denture delivery was recorded as the secondary outcome [23,24]. Masticatory efficiency was evaluated by the volumetric single-sieve method as described by Kapur and Soman [25]. Raw carrots (5 g, hard food) cut into the shape of discs and boiled peanuts (3 g, soft food) were used as the natural test foods (Figure 2).

**Figure 3:**
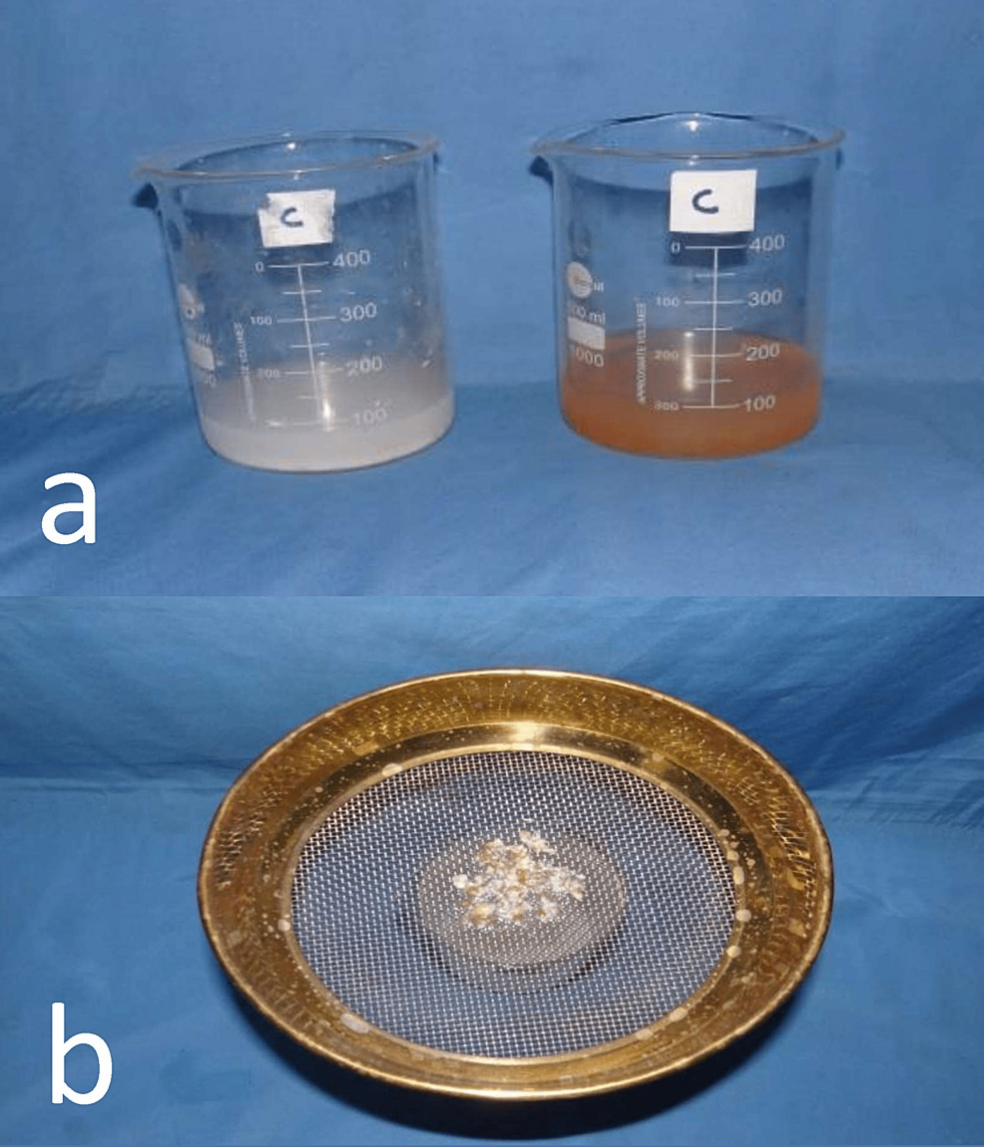
Masticated food collected in a beaker and filtered with sieves a) Collection of masticated food, and b) filtration using test sieves.

Patients were asked to chew the food for about 20 chewing strokes for boiled peanuts and 40 chewing strokes for raw carrots. Masticated food and rinsed water were collected in a beaker and sieved in U.S. standard mesh sieve No.10 (ASTM -10, Manikarn Scientific Works, Haryana) for peanut and U.S. standard mesh sieve No.5 (ASTM -5, Manikarn Scientific Works, Haryana) for the raw carrot (Figure 3).

**Figure 4:**
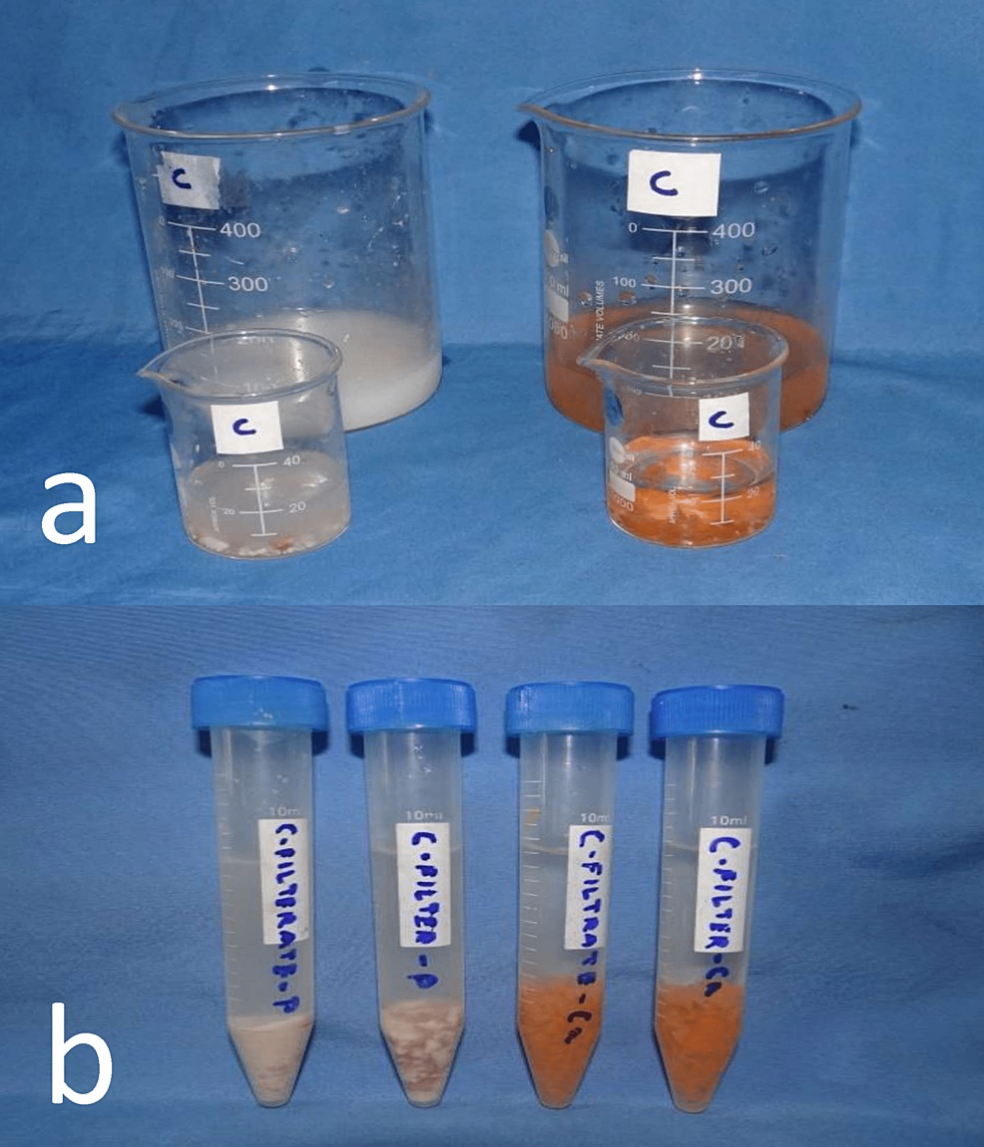
Collection of filter and filtrate followed by centrifugation a) Filter and filtrate in the beaker, and b) test food after centrifugation.

Particles filtered in the sieves were also cleaned with 20 ml of water and collected in a beaker. The two filtrates were centrifuged (Remi R-8C, Remi Elektrotechnik Ltd, India) for three minutes at 1,500 rpm, and the volume of the filtrates was evaluated (Figure 4). Masticatory efficiency was recorded as the percentage of the volume of test food passing through the sieve to the total volume of food particles recovered [25].

An abbreviated version of the Oral Health Impact Profile questionnaire for edentulous patients (OHIP-EDENT) was used for patient satisfaction scores [26]. The abbreviated version had 19 questions which were rearranged into four domains, namely masticatory-related complaints, psychological discomfort and disability, social disability, and oral pain and discomfort (Table 2). This questionnaire had been tested for internal consistency and reliability and showed a Cronbach’s α of 0.86 or 0.90 [26,27]. Scoring for this questionnaire was done on a scale of 0 to 2 (0 = never; 1 = sometimes; 2 = almost always). The total OHIP-EDENT score was calculated by adding all the responses to the 19 questions (ranging from 0 to 38). Individual domain scores were calculated by adding the response to all the questions in that domain.

**Table 2:** Short version of OHIP-EDENT questionnaire with its reorganized domains This source is from Souza et al. [27]. OHIP-EDENT: Oral Health Impact Profile questionnaire for edentulous patients.

Original domain	Question No.	Short version of the question	New domain
Physical disability	Q1	Interrupts meals	Masticatory-related complaints
Physical pain	Q2	Uncomfortable to eat	
Functional limitation	Q3	Difficulty in chewing	
Physical disability	Q4	Avoids eating	
Psychological discomfort	Q5	Self-conscious	Psychological discomfort and disability
Physical disability	Q6	Unable to eat	
Psychological disability	Q7	Upset	
Psychological discomfort	Q8	Worried	
Psychological disability	Q9	Has been embarrassed	
Social disability	Q10	Irritable with others	Social disability
Social disability	Q11	Less tolerant of others	
Handicap	Q12	Unable to enjoy company	
Social disability	Q13	Avoids going out	
Handicap	Q14	Life unsatisfying	
Functional limitation	Q15	Food catching	Oral pain and discomfort
Physical pain	Q16	Painful aching	
Functional limitation	Q17	Dentures not fitting	
Physical pain	Q18	Uncomfortable dentures	
Physical pain	Q19	Sore spots	

Data analysis

All the data obtained were tested for normality using the Kolmogorov-Smirnov test. Data were analyzed using either unpaired Student’s T-test or Mann-Whitney U test for normal and non-normal data, respectively. The gender characteristics of the sample were compared using the Chi-Square test.

## Results

**Figure 5:**
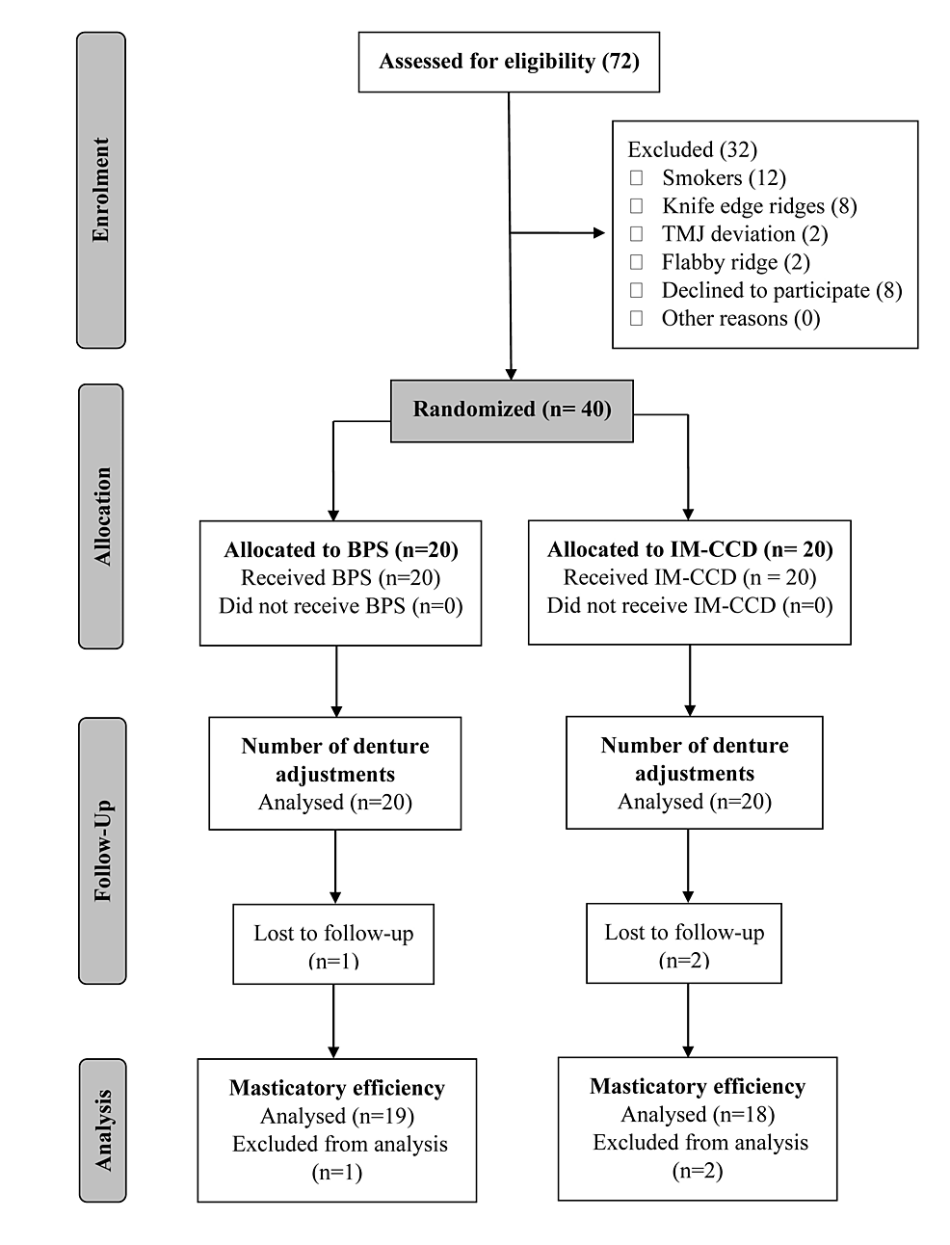
CONSORT flow diagram BPS: biofunctional prosthetic system, IM-CCD: injection-molded conventional complete dentures, TMJ: temporomandibular joint, CONSORT: Consolidated Standards of Reporting Trials.

In total, 72 patients were assessed for eligibility out of which 24 were excluded and eight patients were not willing to participate (Figure 5). After the initial denture adjustments, two patients in the IM-CCD group and one patient in the BPS group were lost to further follow-up. Hence only the secondary outcome data were analyzed for these patients. Primary outcome data of patients who were lost to follow-up were excluded from the analysis. Data obtained for total OHIP-EDENT scores, masticatory efficiency, and denture adjustments were found to be normally distributed. Data obtained for individual domain scores of the OHIP-EDENT questionnaire were not normally distributed. Statistical analysis was done using IBM SPSS statistics software (Version 19, Armonk, NY: IBM Corp).

No significant differences (p>0.05) were found between the two groups for age (Table 3) and gender characteristics (Table 4) of the samples.

**Table 3:** Age characteristics of the sample ^a^Calculated using unpaired Student’s T-test. N: Samples per group, SD: standard deviation, NS: no statistically significant difference between the groups (p>0.05), BPS: biofunctional prosthetic system, IM-CCD: injection-molded conventional complete denture.

Group	N	Age in years (mean ± SD)	Mean difference	95% Confidence interval		t	p
				Lower bound	Upper bound		
BPS	20	60.5 ± 8.22	-1.40	-6.74	3.94	0.53	0.6^a ^(NS)
IM-CCD	20	61.9 ± 8.47					

**Table 4:** Gender characteristics of the sample ^b^Calculated using the Chi-square test. N: samples per group, SD: standard deviation, NS: no statistically significant difference between the groups (p>0.05), BPS: biofunctional prosthetic system, IM-CCD: injection-molded conventional complete denture.

Group	Gender		Chi-square	Degrees of freedom	p
	Male (N)	Female (N)			
BPS	13	7	1.616	1	0.2^b^(NS)
IM-CCD	9	11			

Masticatory efficiency for both peanut (Table 5) and carrot (Table 6) was found to be significantly higher (p<0.05) in the BPS group than in the IM-CCD group.

**Table 5:** Comparison of masticatory efficiency (as a percentage) for soft food (peanut) between BPS and IM-CCD groups ^a^Calculated using unpaired Student’s T-test. N: samples per group, SD: standard deviation, S: statistically significant difference between the groups (p<0.05), BPS: biofunctional prosthetic system, IM-CCD: injection-molded conventional complete denture.

Group	N	Mean ± SD	Mean difference	t value	P	95% CI for difference	
						Lower bound	Upper bound
BPS	19	42.59 ± 11.34	-9.21	-2.64	0.012^a ^(S)	-16.31	-2.12
IM-CCD	18	33.38 ± 9.89					

**Table 6:** Comparison of masticatory efficiency (as a percentage) for hard food (carrot) between BPS and IM-CCD groups ^a^Calculated using unpaired Student’s T-test. N: samples per group, SD: standard deviation, S: statistically significant difference between the groups (p<0.05), BPS: biofunctional prosthetic system, IM-CCD: injection-molded conventional complete denture.

Group	N	Mean ± SD	Mean difference	t value	P	95% CI for difference	
						Lower bound	Upper bound
BPS	19	43.93 ± 12.34	-13.85	-3.98	0.00^a ^(S)	-20.92	-6.77
IM-CCD	18	30.08 ± 8.55					

Although the total OHIP-EDENT score for the IM-CCD group was higher than the BPS group (Table 7), the difference was found to be statistically insignificant (p>0.05).

**Table 7:** Comparison of total OHIP-EDENT scores between BPS and IM-CCD groups ^a^Calculated using unpaired Student’s T-test. N: samples per group, SD: standard deviation, NS: no statistically significant difference between the groups (p>0.05), BPS: biofunctional prosthetic system, IM-CCD: injection-molded conventional complete denture.

Group	N	Mean ± SD	Mean Difference	Standard error of difference	t value	p	95% CI for difference	
							Lower bound	Upper bound
BPS	19	2.68 ± 1.92	-0.49	0.61	-0.79	0.43^a ^(NS)	-1.73	0.75
IM-CCD	18	3.17 ± 1.79						

No significant differences (p>0.05) in the mean scores were observed between the two groups for the individual domain scores (Table 8).

**Table 8:** Comparison of individual domain scores between BPS and IM-CCD groups ^b^Calculated using the Mann-Whitney U test. N: samples per group, SD: standard deviation, NS: no statistically significant difference between the groups (p>0.05), BPS: biofunctional prosthetic system, IM-CCD: injection-molded conventional complete denture.

Domains	BPS (N=19)	IM-CCD (N=18)	U-value	Z-score	p
Masticatory-related complaints	1.21 ± 1.08	1.33 ± 1.08	164.5	-0.182	0.86^b ^(NS)
Psychological discomfort and disability	0.53 ± 0.61	0.44 ± 0.70	153	0.53	0.59^b ^(NS)
Social disability	1.26 ± 0.87	1.33 ± 0.77	163.5	-0.21	0.83^b ^(NS)
Oral pain and discomfort	0.95 ± 1.35	1.39 ± 1.29	128	-1.29	0.19^b ^(NS)

No significant differences (p>0.05) were observed between the groups for the number of denture adjustments done (Table 9).

**Table 9:** Comparison of secondary outcomes between BPS and IM-CCD groups ^a^Calculated using unpaired Student’s T-test. N: Samples per group, SD: standard deviation, NS: no statistically significant difference between the groups (p>0.05), BPS: biofunctional prosthetic system, IM-CCD: injection-molded conventional complete denture.

Secondary outcome	Denture adjustments			
	Maxilla		Mandible	
	BPS (N=20)	IM-CCD (N=20)	BPS (N=20)	IM-CCD (N=20)
Total	32	41	47	59
Mean ± SD	1.6 ± 0.94	2.05 ± 1.00	2.35 ± 1.09	2.95 ± 1.1
Mean difference	0.45		0.6	
t value	1.47		1.73	
p	0.15^a ^(NS)		0.09^a ^(NS)	
95% CI: lower bound	-0.17		-0.10	
95% CI: upper bound	1.07		1.30	

## Discussion

In our study, we found that there was no significant difference between the BPS group and the IM-CCD group in terms of patient satisfaction scores or the number of post-insertion denture adjustments (p>0.05). However, the masticatory efficiency of patients wearing BPS dentures was better than that of patients wearing IM-CCD (p<0.05). Hence, our null hypothesis was rejected.

One of the key differences between BPS and IM-CCD dentures was the method of teeth arrangement followed. For the BPS dentures, teeth arrangement was done using a template. The template incorporates the curves of Wilson and Spee and thus facilitates the arrangement of teeth along these curves [11,16,17]. Functional cusps of the posterior denture teeth were arranged such that they contact the template. In the IM-CCD group, the arrangement of teeth was not done using any template. Hence the curves of Wilson and Spee were more flattened in the IM-CCD group. Osborn [28] demonstrated that forward tilting of the mandibular posterior teeth conforming to the curve of Spee increases the crush/shear ratio of the force produced on food between the posterior molars. This happens because the long axes of the teeth are placed parallel to the superficial fibers of the masseter muscle which maximizes the chewing efficiency during muscle function [28]. When the curve of Wilson is flattened, lingual cusps of the mandibular posterior teeth are placed higher than the buccal cusps, and the thrusting of food by the tongue to the occlusal table is impaired. Similarly, when the buccal cusps of the maxillary posterior teeth are placed lower than the palatal cusps, the thrust of food to the occlusal table by the buccinator muscle is impaired [29]. The reduced masticatory efficiency observed in the IM-CCD group could be due to differences in teeth arrangement between the two groups. However, this effect of the curve of Spee and Wilson on the masticatory efficiency requires further investigation.

The other difference in clinical protocol between the BPS and IM-CCD dentures was the method of recording centric relation. In the BPS dentures, the centric record was made using the “Gnathometer M” which is a form of an intra-oral gothic arch tracer. In the IM-CCD group, maxillomandibular records were made without any tracers. Bite registration material was the same in both groups. The accuracy of different methods of recording centric relation and their effect on complete denture function is still controversial. Some studies report no significant differences in condylar positions obtained using different methods for recording centric relation. Keshvad and Winstanley [30] claim that the guided methods of chin point guidance and bilateral manipulation were more accurate than gothic arch tracings. However, few other reports claim the superiority of the intraoral tracing/gothic arch tracing method over the guided methods [12,13]. Thus, it could be suggested that the use of Gnathometer M in the BPS group might have resulted in more accurate centric records than in the IM-CCD group resulting in better function.

In our study, we did not find any difference in the total OHIP-EDENT scores or the domain scores between IM-CCD and BPS dentures. Our results for patient satisfaction scores are identical to the findings of Matsuda et al. [18]. Although the masticatory efficiency of patients with BPS was better than those with IM-CCD, the domain scores of masticatory-related complaints were not different between the two groups. It has been shown that patient satisfaction scores of patients with fair or moderate ridge forms were not affected by occlusal schemes, posterior tooth forms, impression techniques, or processing methods. Rather, patient satisfaction was found to be more influenced by the stability of the mandibular denture [10].

Matsuda et al. [18] reported that the BPS dentures required fewer denture adjustments compared to conventional complete dentures (CCD). In their cross-over trial, the conventional dentures were processed using cold cure acrylic resin, while BPS dentures were processed using the injection molding system. In our study, both BPS dentures and IM-CCD were processed using the injection molding technique. This could be the reason for the findings of our study. Xhajanka et al. [16] also concluded that the observed differences in decubitus between BPS and conventional dentures were due to differences in processing methods used in their study.

Limitations of our study include the short follow-up period (six weeks), small sample size, and inclusion of only ideal or minimally compromised patients. Hence, studies with longer observation periods, larger sample sizes, and stratification according to the severity of edentulousness are required.

## Conclusions

Within the limitations of the trial, it can be concluded that the BPS dentures significantly improved the masticatory efficiency for both hard (carrots) and soft (peanuts) foods compared to the IM-CCD. However, there was no difference between the masticatory-related complaints domain scores between the two dentures. No significant differences were found between BPS dentures and IM-CCD with respect to overall patient satisfaction scores or post-insertion denture adjustments.
